# Pragmatic randomized trial evaluating pre-operative aqueous antiseptic skin solution in open fractures (Aqueous-PREP): the feasibility of a cluster randomized crossover study

**DOI:** 10.1186/s40814-021-00800-8

**Published:** 2021-03-01

**Authors:** Sheila Sprague, Paige Guyatt, Sofia Bzovsky, Uyen Nguyen, Mohit Bhandari, Lehana Thabane, Brad Petrisor, Herman S. Johal, Jordan Leonard, Shannon Dodds, Franca Mossuto, Robert V. O’Toole, Andrea Howe, Haley K. Demyanovich, Megan Camara, Nathan N. O’Hara, Gerard P. Slobogean, The PREP-IT Investigators, Gerard P. Slobogean, Sheila Sprague, Jeffrey Wells, Mohit Bhandari, Jean-Claude D’Alleyrand, Anthony D. Harris, Daniel C. Mullins, Lehana Thabane, Amber Wood, Gregory J. Della Rocca, Joan Hebden, Lucas Marchand, Lyndsay M. O’Hara, Robert Zura, Michael J. Gardner, Jenna Blasman, Jonah Davies, Stephen Liang, Monica Taljaard, P. J. Devereaux, Gordon H. Guyatt, Diane Heels-Ansdell, Debra Marvel, Jana Palmer, Jeff Friedrich, Nathan N. O’Hara, Ms. Frances Grissom, I. Leah Gitajn, Kyle J. Jeray, Saam Morshed, Robert V. O’Toole, Bradley A. Petrisor, Megan Camara, Franca Mossuto, Manjari G. Joshi, Justin Fowler, Jessica Rivera, Max Talbot, Shannon Dodds, Alisha Garibaldi, Silvia Li, Uyen Nguyen, David Pogorzelski, Alejandra Rojas, Taryn Scott, Gina Del Fabbro, Olivia Paige Szasz, Paula McKay, Andrea Howe, Joshua Rudnicki, Haley Demyanovich, Kelly Little, C. Daniel Mullins, Michelle Medeiros, Eric Kettering, Diamond Hale, Andrew Eglseder, Aaron Johnson, Christopher Langhammer, Christopher Lebrun, Theodore Manson, Jason Nascone, Ebrahim Paryavi, Raymond Pensy, Andrew Pollak, Marcus Sciadini, Yasmin Degani, Haley K. Demyanovich, Katherine Joseph, Brad A. Petrisor, Herman Johal, Bill Ristevski, Dale Williams, Matthew Denkers, Krishan Rajaratnam, Jamal Al-Asiri, Jordan Leonard, Francesc A. Marcano-Fernández, Jodi Gallant, Federico Persico, Marko Gjorgjievski, Annie George, Roman M. Natoli, Greg E. Gaski, Todd O. McKinley, Walter W. Virkus, Anthony T. Sorkin, Jan P. Szatkowski, Joseph R. Baele, Brian H. Mullis, Lauren C. Hill, Andrea Hudgins, Patrick Osborn, Sarah Pierrie, Eric Martinez, Joseph Kimmel, John D. Adams, Michael L. Beckish, Christopher C. Bray, Timothy R. Brown, Andrew W. Cross, Timothy Dew, Gregory K. Faucher, Richard W. Gurich, David E. Lazarus, S. John Millon, M. Jason Palmer, Scott E. Porter, Thomas M. Schaller, Michael S. Sridhar, John L. Sanders, L. Edwin Rudisill, Michael J. Garitty, Andrew S. Poole, Michael L. Sims, Clark M. Walker, Robert M. Carlisle, Erin Adams Hofer, Brandon S. Huggins, Michael D. Hunter, William A. Marshall, Shea Bielby Ray, Cory D. Smith, Kyle M. Altman, Julia C. Bedard, Markus F. Loeffler, Erin R. Pichiotino, Austin A. Cole, Ethan J. Maltz, Wesley Parker, T. Bennett Ramsey, Alex Burnikel, Michael Colello, Russell Stewart, Jeremy Wise, M. Christian Moody, Stephanie L. Tanner, Rebecca G. Snider, Christine E. Townsend, Kayla H. Pham, Abigail Martin, Emily Robertson, Theodore Miclau, Utku Kandemir, Meir Marmor, Amir Matityahu, R. Trigg McClellan, Eric Meinberg, David Shearer, Paul Toogood, Anthony Ding, Erin Donohue, Tigist Belaye, Eleni Berhaneselase, Alexandra Paul, Kartik Garg, Joshua L. Gary, Stephen J. Warner, John W. Munz, Andrew M. Choo, Timothy S. Achor, Milton L. “ Chip” Routt, Mayank Rao, Guillermo Pechero, Adam Miller, Jennifer E. Hagen, Matthew Patrick, Richard Vlasak, Thomas Krupko, Kalia Sadasivan, Chris Koenig, Daniel Bailey, Daniel Wentworth, Chi Van, Justin Schwartz, Niloofar Dehghan, Clifford B. Jones, J. Tracy Watson, Michael McKee, Ammar Karim, Michael Talerico, Debra L. Sietsema, Alyse Williams, Tayler Dykes, William T. Obremskey, Amir Alex Jahangir, Manish Sethi, Robert Boyce, Daniel J. Stinner, Phillip Mitchell, Karen Trochez, Andres Rodriguez, Vamshi Gajari, Elsa Rodriguez, Charles Pritchett, Christina Boulton, Jason Lowe, Jason Wild, John T. Ruth, Michel Taylor, Andrea Seach, Sabina Saeed, Hunter Culbert, Alejandro Cruz, Thomas Knapp, Colin Hurkett, Maya Lowney, Michael Prayson, Indresh Venkatarayappa, Brandon Horne, Jennifer Jerele, Linda Clark, Francesc Marcano-Fernández, Montsant Jornet-Gibert, Laia Martínez-Carreres, David Martí-Garín, Jorge Serrano-Sanz, Joel Sánchez-Fernández, Matsuyama Sanz-Molero, Alejandro Carballo, Xavier Pelfort, Francesc Acerboni-Flores, Anna Alavedra-Massana, Neus Anglada-Torres, Alexandre Berenguer, Jaume Cámara-Cabrera, Ariadna Caparros-García, Ferran Fillat-Gomà, Ruben Fuentes-López, Ramona Garcia-Rodriguez, Nuria Gimeno-Calavia, Guillem Graells-Alonso, Marta Martínez-Álvarez, Patricia Martínez-Grau, Raúl Pellejero-García, Ona Ràfols-Perramon, Juan Manuel Peñalver, Mònica Salomó Domènech, Albert Soler-Cano, Aldo Velasco-Barrera, Christian Yela-Verdú, Mercedes Bueno-Ruiz, Estrella Sánchez-Palomino, Ernesto Guerra, Yaiza García, Nicholas M. Romeo, Heather A. Vallier, Mary A. Breslin, Joanne Fraifogl, Eleanor S. Wilson, Leanne K. Wadenpfuhl, Paul G. Halliday, Darius G. Viskontas, Kelly L. Apostle, Dory S. Boyer, Farhad O. Moola, Bertrand H. Perey, Trevor B. Stone, H. Michael Lemke, Mauri Zomar, Ella Spicer, Chen “ Brenda” Fan, Kyrsten Payne, Kevin Phelps, Michael Bosse, Madhav Karunakar, Laurence Kempton, Stephen Sims, Joseph Hsu, Rachel Seymour, Christine Churchill, Claire Bartel, Robert Miles Mayberry, Maggie Brownrigg, Cara Girardi, Ada Mayfield, Robert A. Hymes, Cary C. Schwartzbach, Jeff E. Schulman, A. Stephen Malekzadeh, Michael A. Holzman, Lolita Ramsey, James S. Ahn, Farhanaz Panjshiri, Sharmistha Das, Antoinisha D. English, Sharon M. Haaser, Jaslynn A. N. Cuff, Holly Pilson, Eben A. Carroll, Jason J. Halvorson, Sharon Babcock, J. Brett Goodman, Martha B. Holden, Debra Bullard, Wendy Williams, Thomas F. Higgins, Justin M. Haller, David L. Rothberg, Ashley Neese, Mark Russell, Marcus Coe, Kevin Dwyer, Devin S. Mullin, Clifford A. Reilly, Peter DePalo, Amy E. Hall, Marilyn Heng, Mitchel B. Harris, R. Malcolm Smith, David W. Lhowe, John G. Esposito, Mira Bansal, Patrick F. Bergin, George V. Russell, Matthew L. Graves, John Morellato, Heather K. Champion, Leslie N. Johnson, Sheketha L. McGee, Eldrin L. Bhanat, Samir Mehta, Derek Donegan, Jaimo Ahn, Annamarie Horan, Mary Dooley, Ashley Kuczinski, Ashley Iwu, David Potter, Robert VanDemark, Branden Pfaff, Troy Hollinsworth, Michael J. Weaver, Arvind G. von Keudell, Michael F. McTague, Elizabeth M. Allen, Todd Jaeblon, Robert Beer, Mark J. Gage, Rachel M. Reilly, Cindy Sparrow

**Affiliations:** 1 Division of Orthopedic Surgery, Department of Surgery, McMaster University, Hamilton, Ontario Canada; 2 Department of Health Research Methods, Evidence, and Impact, McMaster University, Hamilton, Ontario Canada; 3 R Adams Cowley Shock Trauma Center, Department of Orthopedics, University of Maryland School of Medicine, Baltimore, MD USA

**Keywords:** Pilot study, Feasibility, Open fractures, Aqueous antiseptic skin solution, Surgical site infection, Cluster crossover

## Abstract

**Background:**

Preoperative antiseptic skin solutions are used prior to most surgical procedures; however, there is no definitive research comparing infection-related outcomes following use of the various solutions available to orthopedic trauma surgeons. The objective of this pilot study was to test the feasibility of a cluster randomized crossover trial that assesses the comparative effectiveness of a 10% povidone-iodine solution versus a 4% chlorhexidine gluconate solution for the management of open fractures.

**Methods:**

Two orthopedic trauma centers participated in this pilot study. Each of these clinical sites was randomized to a starting solution (povidone-iodine solution or chlorhexidine gluconate) then subsequently crossed over to the other treatment after 2 months. During the 4-month enrollment phase, we assessed compliance, enrollment rates, participant follow-up, and accurate documentation of the primary clinical outcome. Feasibility outcomes included (1) the implementation of the interventions during a run-in period; (2) enrollment of participants during two 2-month enrollment phases; (3) application of the trial interventions as per the cluster randomization crossover scheme; (4) participant follow-up; and (5) accurate documentation of the primary outcome (surgical site infection). Feasibility outcomes were summarized using descriptive statistics reported as means (standard deviation) or medians (first quartile, third quartile) for continuous variables depending on their distribution and counts (percentage) for categorical variables. Corresponding 95% confidence intervals (CIs) were also reported.

**Results:**

All five of the criteria for feasibility were met. During the run-in phase, all 18 of the eligible patients identified at the two clinical sites received the correct cluster-assigned treatment. A total of 135 patients were enrolled across both sites during the 4-month recruitment phase, which equates to 92% (95% CI 85.9 to 96.4%) of eligible patients being enrolled. Compliance with the assigned treatment in the pilot study was 98% (95% CI 93.5 to 99.8%). Ninety-eight percent (95% CI 93.5 to 99.8%) of participants completed the 90-day post-surgery follow-up and the primary outcome (SSI) was accurately documented for 100% (95% CI 96.6 to 100.0%) of the participants.

**Conclusions:**

These results confirm the feasibility of a definitive study comparing antiseptic solutions using a cluster randomized crossover trial design. Building upon the infrastructure established during the pilot phase, a definitive study has been successfully initiated.

**Trial registration:**

ClincialTrials.gov, number NCT03385304. Registered December 28, 2017.

## Key messages

- Given that there is limited research addressing the comparative outcomes associated with various types of antiseptics in open fracture surgery and that Aqueous-PREP follows a cluster randomized crossover design, which is novel in the field of orthopedic trauma surgery, a pilot study was conducted.
- All feasibility criteria were met in the pilot study:
  - ○ Enrollment of 92% of open fracture patients during the enrollment periods
  - ○ 98% of participants receiving the treatment as per the cluster randomization on their initial surgery
  - ○ Participant follow-up of 98% at 90-day post-fracture surgery
  - ○ Accurate documentation of 100% of the primary outcome (SSI)
- Building upon the infrastructure, developed, and momentum and insights gained through the pilot study, we were able to successfully transition directly into the definitive trial phase of the Aqueous-PREP trial.

## Introduction

Sterile technique and pre-operative skin cleaning with antiseptic solutions are vital components of peri-operative care for open fracture patients as they decrease surgical site infections (SSI) [1–4]. Antiseptic solutions, when applied to the skin immediately prior to surgery, are able to destroy bacteria and also diminish the amount of native skin flora and therefore are thought to reduce surgical site infection [1–4]. Skin preparation solutions are either alcohol or aqueous-based, and commonly include either an iodophor or chlorhexidine-based active ingredient.

Despite the recognized importance of applying skin preparation solutions immediately prior to surgery, there is limited research addressing the comparative outcomes associated with various types of antiseptics in open fracture surgery. Much of the existing evidence comes from research conducted in other surgical contexts. The limitations of generalizing results from other surgical settings to open fracture wound management is widely recognized, as the risk of SSI is substantially greater in fracture patients [4]. Fracture patients experience a higher potential for contamination for several reasons, including soft tissue damage following an injury, diminished blood flow to the injured limb if a tourniquet is used, the limited opportunity for additional prophylactic skin care due to the emergent nature of fracture surgery, and the risk related to inserting a metal implant that may promote bacterial biofilm growth [5].

The limited research that has investigated surgical skin preparation effectiveness in open fracture management found that povidone-iodine (an aqueous iodophor solution) could potentially provide increased protection in comparison to chlorhexidine; however, the results suggest that iodophor may reduce the odds of infection by 31% or, conversely, increase it up to 12% [6]. Thus, while there are certain chemical properties of povidone-iodine that could potentially allow it to block infection more effectively than CHG [7], it is unknown whether it should be the solution of choice for orthopedic surgeons tending to open fractures.

Given these uncertainties, the PREP-IT Investigators developed the protocol for the Aqueous-PREP (Pragmatic Randomized Trial Evaluating Pre-Operative Aqueous and Antiseptic Skin Solution in Open Fractures) trial, which aims to compare the effectiveness of an aqueous pre-operative antiseptic preparation with 10% povidone-iodine (an iodophor solution) versus 4% CHG for the management of open fractures. A ranked order for assessing effectiveness will be used, with SSI as the primary comparison and unplanned fracture-related reoperations as the secondary comparison. Aqueous-PREP follows a cluster randomized crossover design, which is novel in the field of orthopedic trauma surgery [5]. We selected cluster randomized crossover design over the parallel randomized controlled trial design to facilitate participant enrollment and to reduce the risk of treatment contamination [8, 9]. Due to the novel nature of the design, we conducted a pilot study prior to initiating the Aqueous-PREP trial, with a primary objective to prove the feasibility of comparing these two solutions using cluster randomized crossover trial.

## Methods

Two orthopedic trauma centers participated in this pilot study, one in Canada and one in the USA. Each of these clinical sites was randomized to a starting surgical preparation solution (povidone-iodine solution or chlorhexidine gluconate). After a run-in period and a 2-month enrollment period, they crossed over to the other treatment for an additional 2 months of enrollment. Participants were followed for 90-day post-fracture fixation surgery. Feasibility outcomes were assessed over the duration of the pilot study. The master protocol followed during the implementation of the Aqueous-PREP trial has been published [5], and the objectives and methods are described in detail within this manuscript. The trial was registered with ClincialTrials.gov, number NCT03385304. It was approved by the Hamilton Integrated Research Ethics Board (#4336) and the Advarra Institutional Review Board (formerly Chesapeake Institutional Review Board) (#Pro00023709).

### Randomization and the run-in phase

The order of treatment allocation for each clinical site was randomly assigned by personnel at the Center for Evidence-Based Orthopedics (CEO) Methods Center using a computer-generated randomization table. Prior to initiating patient recruitment, each clinical site completed a 1-month run-in phase. During this time, the sites began using their assigned pre-operative antiseptic skin solution for eligible open fracture surgeries. This run-in period was implemented to ensure that acceptable compliance was achieved before initiating participant enrollment. Compliance was monitored by Methods Center by reviewing data submitted by study personnel at each clinical site.

### Enrollment phases

Following the initial run-in phase, participant recruitment began. Each clinical site continued to use their previously assigned surgical antiseptic skin solution for all eligible open fracture surgeries over an approximate 2-month enrollment period. Participants received the allocated treatment solution for their initial fracture management surgery, as well as for repeat planned surgeries, even if the repeat surgery fell within the subsequent enrollment period that used the non-allocated solution. After the first enrollment period was complete, the sites crossed over to the opposite study solution and moved directly into the second 2-month enrollment phase. No run-in phase was implemented at the time of crossover to the second solution.

### Eligibility criteria

Eligible patients were adult men and women ages 18 or older with an open fracture of the appendicular skeleton, who had received or would receive definitive fracture treatment with surgical implant(s). Patients’ open fracture wound management was required to include formal surgical debridement within 72 h of their injury, with surgeries being performed by a participating surgeon or delegate. All patients or their proxy were required to provide informed consent, and patients were required to be enrolled within 3 weeks of their fracture.

### Clinical outcomes and participant follow-up

The primary outcome was SSI at 90-day post-fracture surgery. Participants were assessed during their hospital stay, and at 6 weeks and 90-day post-fracture surgery.

### Pilot study outcomes and success criteria

The primary feasibility outcome was a composite outcome of five equally weighted factors. To consider the trial “feasible,” all five factors had to be determined to be feasible. These five factors were as follows:

(1) The implementation of the trial intervention during the run-in period (defined as >90% of eligible patients receiving the allocated antiseptic solution during the run-in phase)

(2) Enrollment of participants across the two planned 2-month enrollment phases at two centers (defined as enrollment of >75% of open fracture patients over each of the two 2-month enrollment periods)

(3) Application of the trial interventions as per the cluster randomization crossover scheme (defined as >95% of participants receiving the treatment as per the cluster randomization on their initial surgery)

(4) Achievement of high rates of participant follow-up (defined as participant follow-up of >95% at 90-day post-fracture surgery)

(5) Accurate documentation of the primary outcome (surgical site infection (SSI) defined as less than 5% error rate in the documentation of the primary outcome).

A definitive Aqueous-PREP cluster randomized crossover trial would be considered feasible if the clinical sites participating in the pilot phase collectively reached all five of these thresholds.

### Statistical analysis

#### Sample size

Since the feasibility objectives in our pilot study did not lend themselves to traditional quantitative sample size calculations, we selected a sample size of 2 clinical sites and an enrollment period of 4 months. This enrollment period allowed each clinical site to enroll patients into each of the two treatment groups, and we estimated that between 60 and 100 participants would be enrolled across the two sites. This approach allowed us to assess the feasibility of successfully implementing a large definitive trial.

#### Statistical analysis of outcomes

We followed the CONSORT extensions to pilot and cluster trials when reporting the results of this pilot study [6, 8]. Participant demographic characteristics, fracture characteristics, and feasibility outcomes were summarized using descriptive statistics reported as means (standard deviation (SD)) or medians (first quartile, third quartile) for continuous variables depending on their distribution and counts (percentage) for categorical variables. Corresponding 95% confidence intervals (CIs) were also reported. All data analyses were conducted using R (version 4.0.0, R Foundation for Statistical Computing, Vienna, Austria).

## Results

### Feasibility criteria 1: Run-in phase

**Fig. 1:**
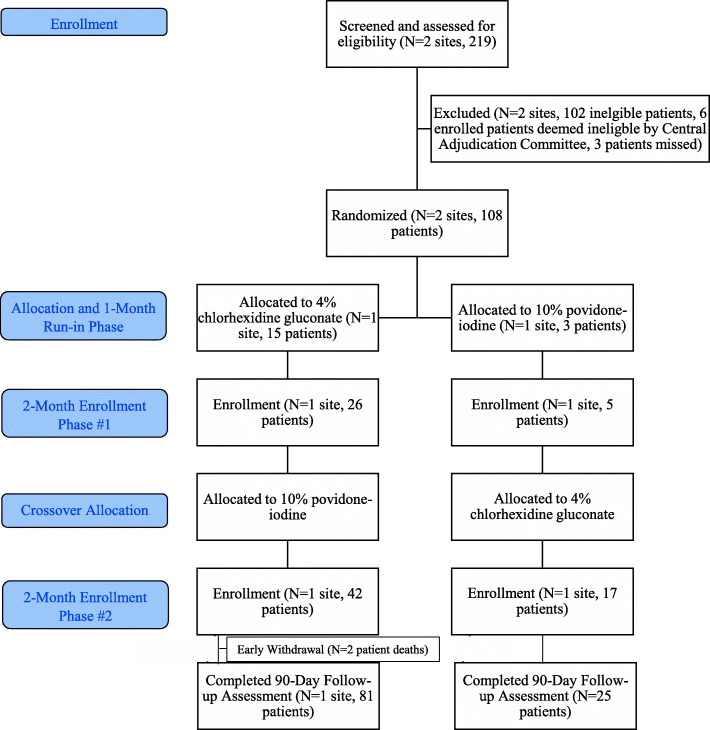
Flow diagram

Two orthopedic trauma centers participated in the Aqueous-PREP pilot trial: Hamilton Health Sciences General Site (HGH) in Hamilton, Ontario, Canada, and the R Adams Cowley Shock Trauma Center (STC) in Baltimore, MD, USA. These pilot sites were randomized to one of the two skin preparation solutions and completed successful run-in phases. During the run-in phase, all eligible patients at both sites were treated with the appropriate surgical skin preparation solutions; STC prepped 15 out of 15 patients correctly and HGH prepped 3 out of 3 patients correctly for a total of 18 patients. After completing a successful run-in period, the sites began patient recruitment (Fig. 1).

### Feasibility criteria 2: Participant enrollment

Over the course of the two 2-month enrollment phases (April 3, 2018 to August 21, 2018), STC screened 167 patients, 76 of which were ineligible and excluded. Ninety-one screened patients were eligible, of which 83 (91%) were enrolled. Over the course of the two 2-month enrollment phases (March 12, 2018 to July 17, 2018), HGH screened 52 patients, 26 of which were ineligible and excluded. Twenty-six screened patients were eligible, of which 25 (96%) were enrolled. The reasons for exclusion are listed in Table 1. Overall, 92% (95% CI 85.9 to 96.4%) of eligible patients at both sites were enrolled during the pilot phase, for a total of 108 participants. A total of 3 patients were missed and 6 patients were deemed ineligible by the Central Adjudication Committee (Table 1). Less than 1% of eligible patients did not provide informed consent. Consequently, enrollment rates were well above the established threshold of 75% of open fracture patients.

**Table 1:** Reasons for exclusion

Reasons for exclusion*	Number of patients excluded during screening ***n***=102	Number of patients excluded following adjudication ***n***=6
Did not receive or will not receive definitive fracture treatment with a surgical implant(s) (e.g., internal fixation, external fixation, joint prosthesis), *n* (%)	33 (32.4)	2 (33.3)
Open fracture wound management did not include a formal surgical debridement within 72 h of their injury, *n* (%)	21 (20.6)	2 (33.3)
Likely problems, in the judgment of the study personnel, with maintaining follow-up, *n* (%)	16 (15.7)	N/A
Incarceration, *n* (%)	8 (7.8)	N/A
Did not provide informed consent, *n* (%)	7 (6.9)	N/A
Unable to obtain informed consent due to language barriers, *n* (%)	6 (5.9)	N/A
Received previous surgical debridement or management of their open fracture(s) at a non-participating hospital or clinic, *n* (%)	4 (3.9)	N/A
Expected injury survival of less than 90 days, *n* (%)	4 (3.9)	N/A
Open fracture(s) managed outside of the participating orthopedic service (e.g., hand fracture managed by plastic surgeon), *n* (%)	1 (1.0)	N/A
Burns at the fracture site, *n* (%)	1 (1.0)	N/A
Not enrolled within 3 weeks of their fracture, *n* (%)	1 (1.0)	1 (16.7)
Patient enrolled during run-in phase	N/A	1 (16.7)

*Please note that some patients were excluded for more than one reason. We present one reason per patient based on order of reason for exclusion in table

The majority of participants were male (*n*=81, 75.0%) and White (*n*=68, 63.0%) with an average age of 42 years (SD 17 years). Ninety-eight of the patients sustained one eligible open fracture (90.7%) while 10 sustained two or three eligible open fractures (9.3%). Thirty-nine of all 120 fractures (32.5%) were of the upper extremities, while 81 (67.5%) were lower extremity fractures, the most common of which were tibial and tibial shaft fractures (*n*=49 (40.8% of all 120 fractures)). According to the Gustilo Classification of Fracture system, 22 fractures were type I (18.3%), 36 were type II (30.0%), 41 were type IIIA (34.2%), 13 were type IIIB (10.8%), and 8 were type IIIC (6.7%). Further details on participant demographics and fracture and injury characteristics are provided in Table 2.

**Table 2:** Participant demographics and fracture characteristics

Characteristic	Total ***n***=108
Age, mean (SD) years	41.9 (17.1)
Gender, *n* (%)	
Female	27 (25.0)
Male	81 (75.0)
BMI, (kg/m^2^), *n* (%)	
Underweight <18.5	4 (3.7)
Normal weight 18.5-24.9	37 (34.3)
Overweight 25-29.9	39 (36.1)
Obese 30-39.9	21 (19.4)
Morbidly obese ≥40	7 (6.5)
Ethnicity, *n* (%)	
White	68 (63.0)
Black	24 (22.2)
Hispanic/Latino	8 (7.4)
East Asian	2 (1.9)
Middle Eastern	5 (4.6)
Indigenous	1 (0.9)
Employment prior to injury, *n* (%)	69 (63.9)
Highest level of education completed, *n* (%)	
8th grade or less	1 (1.0)
9th to 12 grade, no diploma	10 (9.3)
General education diploma or high school graduate	39 (36.1)
Some college, no degree	23 (21.3)
Associates degree (2-year degree)	8 (7.4)
Bachelors/college degree	12 (11.1)
Some graduate work, no degree	1 (0.9)
Graduate degree	7 (6.5)
Professional degree	3 (2.8)
Prefer not to answer	4 (3.7)
Smoking history, *n* (%)	
No, never smoked	42 (38.9)
Yes, previously smoked	22 (20.4)
Yes, smokes currently	44 (40.7)
Number of open fractures, *n* (%)	
1	98 (90.7)
2	8 (7.4)
3	2 (1.9)
Location of fracture, *n* (%)	*n*=120 fractures among 108 patients
Clavicle	1 (0.8)
Femur/femoral shaft	15 (12.5)
Fibula	2 (1.7)
Foot/ankle	14 (11.7)
Hand	7 (5.8)
Humerus/humeral shaft	13 (10.8)
Patella	1 (0.8)
Radius/radial shaft	10 (8.3)
Tibia/tibial shaft	49 (40.8)
Ulna/ulnar shaft	8 (6.7)
Gustilo classification of fracture, *n* (%)	*n*=120 fractures among 108 patients
I	22 (18.3)
II	36 (30.0)
IIIA	41 (34.2)
IIIB	13 (10.8)
IIIC	8 (6.7)

Percentages represent proportion out of total number of fractures by treatment group. Some participants sustained multiple fractures

*BMI* body mass index, *SD* standard deviation

### Feasibility criteria 3: Treatment received as per cluster randomization

During the two 2-month enrollment phases, contamination rates were closely monitored. Ninety-seven percent (81/83) of participants at STC were prepped correctly during their initial surgery. At HGH, 100% (25/25) of participants were prepped correctly during their initial surgery. The two contaminations that did occur at STC were due to surgeon error. Overall, 98% (106/108) (95% CI 93.5 to 99.8%) of participants across both pilot sites received the correct preparation solution during their initial surgery. Collectively, the two sites met the 95% feasibility threshold for correct treatment (Table 3).

**Table 3:** Feasibility outcomes

Feasibility outcomes	Feasibility criteria	Pilot study results ***n*** (%)	Feasibility criteria met
Implementation of the two trial interventions during the 1-month run-in period, *n* (%)	At least 15 eligible open fracture patients with >90% of eligible patients receiving the allocated antiseptic solution or a minimum of 1 month in duration	Total: 18/18 (100%) STC: 15/15 (100%) HGH: 3/3 (100%)	Yes
Enrollment of participants across the two planned 2-month enrollment phases, *n* (%)	Enrollment of >75% of open fracture patients over each of the two 2-month enrollment periods	Total: 108/117 (92%) STC: 83/91 (91%) HGH: 25/26 (96%)	Yes
Participants receiving trial interventions as per the cluster randomization crossover scheme, *n* (%)	>95% of participants receiving the treatment as per the cluster randomization on their initial surgery	Total: 106/108 (98%) STC: 81/83 (97%) HGH: 25/25 (100%)	Yes
Rates of Participant Follow-up, n (%)	Participant follow-up of >95% at 90-day post-fracture surgery.	Total: 106/108 (98%) STC: 81/83 (97%) HGH: 25/25 (100%)	Yes
Accurate documentation of the primary outcome, *n* (%)	Accurate documentation of >95% of the primary outcome (SSI) (i.e., less than 5% error rate in the documentation of the primary outcome).	Total: 100%	Yes

### Feasibility criteria 4: Participant follow-up

Participant follow-up visits were also completed as per protocol as part of the pilot study. Ninety-seven percent (81/83) of participants at STC completed follow-up at 90-day post-fracture surgery. Two of the 83 enrolled participants at STC died prior to completing follow-up. One hundred percent (25/25) of participants at HGH completed follow-up at 90-day post-fracture surgery. Overall, 98% (106/108) (95% CI 93.5 to 99.8%) of participants across both pilot sites completed follow-up at 90-day post-fracture surgery, which exceeded the 95% threshold for acceptable follow-up.

### Feasibility criteria 5: Documentation of the primary outcome

The primary outcome (SSI) was documented 100% (95% CI 96.6 to 100.0%) accurately across both pilot sites. During the pilot trial, the CEO Methods Center conducted remote and on-site monitoring to ensure the pilot sites reported all events. As an error rate of less than 5% in the documentation of the primary outcome was required, the last feasibility criterion was met.

## Discussion

The need for a definitive trials comparing preoperative antiseptic skin solutions is based on the conflicting results from existing trials, as well as the lack of research that compares antiseptic solutions in a specifically orthopedic surgical setting [10]. These inconsistent results leave the optimal antiseptic solution in doubt; in addition, results may differ across surgical settings. The risk of SSI is substantially greater in certain fracture populations (open fractures, closed lower extremity fractures, and pelvic fractures) due to the soft tissue trauma, wound contamination in open fractures, the increased risk of local vascular disruption, and the required surgery to fix the broken bones. Furthermore, the emergent nature of fracture surgery means that patients are unable to undergo other prophylactic skin care, such as CHG bathing, which is rendered to elective cases to reduce SSI. Additionally, the timing of prophylactic antibiotics may also fall beyond the recommended windows due to delays in getting to hospital; therefore, local antisepsis may become even more critical. The PREP-IT program includes two cluster randomized crossover trials which will definitely determine the optimal surgical preparation solution for this population. The Aqueous-PREP trial has been designed to study the effectiveness of a 10% povidone-iodine aqueous based solution versus 4% CHG aqueous-based solution in reducing SSI. The PREPARE (Pragmatic Randomized Trial Evaluating Pre-Operative Alcohol Skin Solutions in Fractured Extremities) trial follows the same design as the Aqueous-PREP trial; however, it compares DuraPrep™ versus ChloraPrep™, which are alcohol-based solutions, in the management of open and closed fractures [5]. Similar to the Aqueous-PREP trial, the primary outcome of the PREPARE trial is SSI and the secondary outcome is unplanned fracture-related reoperations within 12 months [5]. Two sister trials are required as there is a lack of consensus on the use of alcohol-based solutions in open fracture patients.

The Aqueous-PREP pilot trial, implemented at two orthopedic trauma centers, was used to test the feasibility of using a cluster randomized crossover study scheme to fill this gap in the existing research. All of the criteria corresponding to these five feasibility outcomes were met during the pilot phase of the Aqueous-PREP trial. The run-in phase was successful in regard to the number of eligible open-fracture patients received at each orthopedic study center. Although HGH treated only 3 eligible patients, this was anticipated as they are a smaller site in comparison to STC and their run-in period occurred in March when trauma volume is typically very low. During the 4-month enrollment period, 92% of patients across both sites were enrolled. As expected, enrollment at both clinical sites was higher during the second 2-month enrollment period due to seasonal variations in volume of traumatic injuries at both clinical sites. In response to the Central Adjudication Committee’s determination that a number of participants enrolled at STC were ineligible, the team was retrained to ensure that additional enrollment errors would not occur in future. Informed consent was obtained from more than 99% of eligible patients. This high rate of consent is most likely attributable to the fact that the trial design allows for informed consent to take place after the intervention.

Compliance remained high during the run-in phase as well as over the course of the two enrollment periods. No contamination (i.e., the incorrect solution being applied as per cluster randomization) occurred during the run-in phase, and 99% of patients enrolled thereafter received the correct treatment during their initial surgery. Noncompliance rates increased to 5% when accounting for the surgeries of patients who underwent repeat planned surgeries; however, the feasibility threshold of 95% compliance did not apply to these additional procedures. The contaminations that did occur during the intervention phases were due to (1) surgeon error, (2) plastic surgery prepped the participant, and (3) surgery took place at another hospital. Half of the instances of noncompliance occurred within the first 2-month enrollment period and prior to the crossover between treatments. This suggests that the clinical sites’ switch from the use of one antiseptic solution to another did not significantly increase contamination rates, and thus the crossover scheme was largely unproblematic.

Lastly, acceptable rates of patient follow-up and proper documentation of SSI, the primary outcome, were achieved across study sites. Ninety-eight percent of participants completed follow-up at 90-day post-fracture surgery, and the primary outcome was accurately documented for all participants. As such, all the success criteria were met, clearly suggesting that a definitive cluster randomization crossover study comparing preoperative antiseptic skin solutions is feasible.

While the effectiveness of the case report forms (CRFs) used during this pilot study was not an official outcome, this feasibility study allowed for its informal assessment. Following the completion of the pilot phase and prior to initiation of a definitive trial, the Aqueous-PREP CRFs were significantly modified to facilitate straightforward and efficient data collection. The necessity of these changes demonstrates the importance of conducting pilot phases with limited enrollment before initiating large-scale definitive studies, as researchers may encounter protocol issues or areas of improvement not previously anticipated.

## Conclusions

This pilot study, conducted at two clinical sites in two countries, confirmed the feasibility of a definitive study comparing antiseptic solutions in open fractures using a cluster randomized crossover trial design. This pilot represents a critical first step in the conduct of the Aqueous-PREP trial, especially in light of the fact that this trial follows a cluster randomized crossover design, which is novel in the field of orthopedic trauma surgery. Building upon the infrastructure, developed, and momentum and insights gained through the pilot study, we were able to successfully transition directly into the definitive trial phase of the Aqueous-PREP trial and the PREPARE trial.

## Data Availability

All data generated in this study will be analyzed upon completion of the participant follow-up during the definitive phase of the trial and will be published in a journal article in future.

## Abbreviations

CEO: Center for Evidence-Based Orthopedics
CHG: Chlorhexidine gluconate
HGH: Hamilton Health Sciences General Site
SD: Standard deviation
SSI: Surgical site infection
STC: R Adams Cowley Shock Trauma Center

## References

[1] Darouiche RO, Wall MJ, Itani KMF (2010). Chlorhexidine-alcohol versus povidone-iodine for surgical-site antisepsis. N Engl J Med.

[2] Tuuli MG, Liu J, Stout MJ (2016). A randomized trial comparing skin antiseptic agents at cesarean delivery. N Engl J Med.

[3] Swenson BR, Sawyer RG (2010). Importance of alcohol in skin preparation protocols. Infect Control Hosp Epidemiol.

[4] Swenson BR, Hedrick TL, Metzger R, Bonatti H, Pruett TL, Sawyer RG (2009). Effects of preoperative skin preparation on postoperative wound infection rates: a prospective study of 3 skin preparation protocols. Infect Control Hosp Epidemiol.

[5] Slobogean GP, Sprague S, Program of Randomized Trials to Evaluate Pre-operative Antiseptic Skin Solutions in Orthopaedic Trauma (PREP-IT) Investigators (2020). Effectiveness of iodophor vs chlorhexidine solutions for surgical site infections and unplanned reoperations for patients who underwent fracture repair: The PREP-IT master protocol. JAMA Netw Open.

[6] Eldridge SM, Chan CL, Campbell MJ (2016). CONSORT 2010 statement: extension to randomised pilot and feasibility trials. BMJ..

[7] McDonnell G, Russell AD (1999). Antiseptics and disinfectants: activity, action, and resistance. Clin Microbiol Rev.

[8] Campbell MK, Piaggio G, Elbourne DR, Altman DG, CONSORT Group (2012). Consort 2010 statement: extension to cluster randomised trials. BMJ.

[9] Arnup SJ, Forbes AB, Kahan BC, Morgan KE, McKenzie JE (2016). Appropriate statistical methods were infrequently used in cluster-randomized crossover trials. J Clin Epidemiol.

[10] Wade RG, Burr NE, McCauley G, Bourke G, Efthimiou O. The comparative efficacy of chlorhexidine gluconate and povidone-iodine antiseptics for the prevention of infection in clean surgery: a systematic review and network meta-analysis. Ann Surg. 2020. Online ahead of print. 10.1097/SLA.0000000000004076 32773627

